# Increasing engagement with liver disease management across the UK: a follow-up cross-sectional survey

**DOI:** 10.3399/BJGPO.2024.0142

**Published:** 2025-01-15

**Authors:** Helen Jarvis, Charlotte Berry, Jonathan Worsfold, Vanessa Hebditch, Stephen Ryder

**Affiliations:** 1 Population Health Sciences Institute, Faculty of Medical Sciences, Newcastle University, Newcastle, UK; 2 British Liver Trust Policy and Public affairs Officer, Venta Court, Winchester, UK; 3 British Liver Trust Director of Service Delivery, Venta Court, Winchester, UK; 4 British Liver Trust Director of Communications and Policy, Venta Court, Winchester, UK; 5 NIHR Nottingham Biomedical Research Centre at Nottingham University Hospitals NHS Trust, Nottingham, UK

**Keywords:** liver diseases, quality assurance, diagnosis, primary health care

## Abstract

**Background:**

Liver disease is an increasing cause of premature mortality. Early detection of liver disease in primary care gives opportunity to intervene and change outcomes. Engagement in liver disease care by NHS bodies responsible for primary care pathway development could drive improvements. The formation of integrated care systems (ICS) in England provides an opportunity to reassess engagement with liver disease nationally.

**Aim:**

To update the level of engagement with community chronic liver disease management among ICSs and health authorities across the UK.

**Design & setting:**

A cross-sectional follow-up survey to ICS and UK health boards.

**Method:**

Questions used for a previous survey in 2020 were adapted and sent electronically to NHS bodies responsible for health care across the UK, using a freedom of information request. Quantitative analysis was undertaken using Microsoft Excel.

**Results:**

There were 67 responses from 68 possible ICS and health board areas, representing 99% UK coverage. Twenty-seven per cent had a named individual responsible for liver disease. Monitoring of local liver disease health statistics happened in 34% of all UK areas. Comprehensive care pathways were available in *n* = 24/67 (36%) of areas, an increase from 26% in the 2020 survey. Areas with no liver pathways in place fell from 58% to 36% between the two surveys. Regional variations persist, with Wales and Scotland moving towards comprehensive coverage. Almost double the number of areas were making use of transient elastography within community pathways of care, up from 25% to 46%.

**Conclusion:**

The results of this re-survey highlight improvements, but emphasise the need to build on regional success to further reduce inequality in care commissioning.

## How this fits in

Liver disease mortality continues to increase in the UK. A previous survey in 2020 showed poor and variable engagement with primary care liver disease pathways and management by commissioning bodies. This study reports some improvement, but highlights ongoing inequity in care commissioning with unacceptable regional and local differences. The results should be used by clinicians and policymakers to further reduce the postcode lottery' of primary care for liver disease, with a particular focus on engaging the integrated health boards in England.

## Introduction

Liver disease is an increasing cause of mortality in the UK, with a 21% increase in deaths from liver disease between 2019 and 2021.^1^ This is primarily driven by increased consumption of alcohol, and an increased number of people living with obesity and type 2 diabetes (leading to metabolic-dysfunction associated steatotic liver disease [MASLD], formerly known as non-alcohol related fatty liver disease [NAFLD]). Liver disease is usually asymptomatic in the early stages. If it is diagnosed early, lifestyle intervention can halt or reverse disease progression. In the UK, liver disease is now a leading cause of premature mortality (deaths in people aged <75 years).^2^ There are significant regional variations in liver disease outcomes across the UK, including in rates of hospital admission and mortality.^3^ To avoid late presentation of either decompensated cirrhosis or hepatocellular carcinoma (HCC), with associated high mortality rates, earlier detection of liver disease (at the stage of liver fibrosis) in the community is now accepted as vital.

In the past decade a range of liver fibrosis tests have been developed and validated in liver disease.^4^ These may be indirect scores calculated from readily available routine blood tests (for example, Fibrosis-4 index [FIB-4]), specific direct blood biomarkers of fibrosis pathways (for example, enhanced liver fibrosis [ELF] test) or direct measures of liver stiffness (for example, transient elastography). The detection of liver fibrosis allows identification of populations who are at significant risk of developing severe liver disease before liver failure develops. This allows preventive strategies, such as alcohol reduction and weight management, to be targeted.

In 2020, the British Liver Trust, in collaboration with academic partners, carried out and published a cross-sectional survey of all UK commissioning bodies and health authorities,^5^ which revealed huge inequalities in primary care provision for people at risk of liver disease. Only 26% of the UK had comprehensive community pathways for liver disease management in place. Since 2020, primary care commissioning in England has been re-structured, with 42 new integrated care systems (ICSs) incorporating the 135 previous clinical commissioning groups (CCGs), allowing for roll out of pathways and priority setting on wider regional footprints. The British Liver Trust has been campaigning to make early detection of liver disease routine,^6^ including, as part of this, contacting and meeting integrated care boards (ICBs) and devolved nation health boards to promote best practice. NHS England has also had an increased focus on community diagnostics^7^ as well as early detection of cancer, with a specific workstream on early detection of HCC.^8^

These policy initiatives have been running in parallel to an increasing number of research and quality improvement projects across the regions of the UK, aiming to improve the early detection of liver disease.^9^ Serial surveys of access to liver fibrosis testing from a secondary care provider perspective have indicated increased utility of tests to find liver disease at an earlier stage.^10^

The aim of this study was to reassess the levels of engagement with chronic liver disease management among primary care commissioning bodies and health authorities across the UK, and to address the following questions: Has the formation of ICSs (introduced in July 2022) already had an early impact on pathway development in England? As the ICS covers a wider geographic area, could they offer opportunities for the spread of good practice across a region? Is the increase in use of liver fibrosis testing in secondary care mirrored in primary care across the UK, and what progress has there been since 2020?

## Method

A repeat online cross-sectional survey was sent to all UK health bodies in May 2023, questioning commissioning of pathways of care, the use of recommended diagnostic tests, and overall engagement with liver disease management. All ICSs, as well as devolved nation health boards, were included in the survey. The repeat survey was sent using a freedom of information request (or equivalent in the devolved nations) via email. UK legislation mandates provision of information held by an organisation on receipt of a formal request, as per the Freedom of Information Act 2000 (or equivalent in the devolved nations). Public authorities must comply promptly and respond no later than 20 working days following the date of receipt of the request. It was at the discretion of the health body to select the appropriate responder for the survey.

As with the original survey sent out in 2020, the survey covered the following three main areas of engagement: structural processes and staffing capacity; the use of recommended guidelines and diagnostic tools to detect liver disease; and engagement with proactive risk factor-based detection of liver disease.

Original questions from the 2020 survey were repeated with some additional questions to reflect the new commissioning structure in England. The full survey content is available in the Supplementary Information (Supplementary sheet 1). The new questions ensured that we captured difference in the structure of commissioning bodies in England and that differences between 2020 and 2023 were assessed. New questions are indicated with an asterisk in the Supplementary Information.

The additional questions were developed by the British Liver Trust and lead authors, and reviewed by primary care, hepatology, and patient experts. As such, this updated survey was developed and operationalised with equality of input from professionals and public representatives.

Survey responses were collected and entered onto a Microsoft Excel spreadsheet (by CB) and analysed (by HJ) using simple quantitative analysis methods on Microsoft Excel.

## Results

There were 67 responses to the repeat survey out of 68 possible ICS and health board UK areas, representing 99% UK coverage. There was no response from one Scottish health board, NHS Forth Valley. Responders held various roles within their organisation such as head of planned care, associate director of commissioning in elective care, chief medical officer, clinical lead, or GP.

### Structural processes and staffing capacity in place specific to liver disease

Across the UK, just over one-quarter of ICSs and health boards (27%) had a named individual responsible for liver disease within their organisation (up from 20% in the 2020 survey). According to the responses, 34% of all UK areas reported awareness and monitoring of local liver disease health statistics, unchanged from the 2020 survey. In England, monitoring the efficacy and uptake of pathways of care had dropped from 16% to 2% between surveys, while remaining largely unchanged in the devolved nations.

Comprehensive care pathways were available in *n* = 24/67 (36%) of areas, an increase from 26% in the 2020 survey. When combining the availability of comprehensive pathways of care with those areas with either a partial pathway (usually one that was targeted towards responding to abnormal liver blood tests) or a pathway available locally but not across the ICS footprint, *n* = 43/67 (64%) of areas had a care pathway of some sort available to them in this re-survey. This is a reduction in the number of areas with no liver pathways in place, from 58% to 36% between the two surveys.

Regional variations persist. Improvements were made in Wales, which now has 100% coverage of comprehensive pathways of care for liver disease available in all health boards. Scotland has also increased the coverage of comprehensive primary care pathways, from 42% to 69% of health boards. Northern Ireland did not report a single comprehensive primary care pathway for liver disease across any health trust.

**Figure 1.**
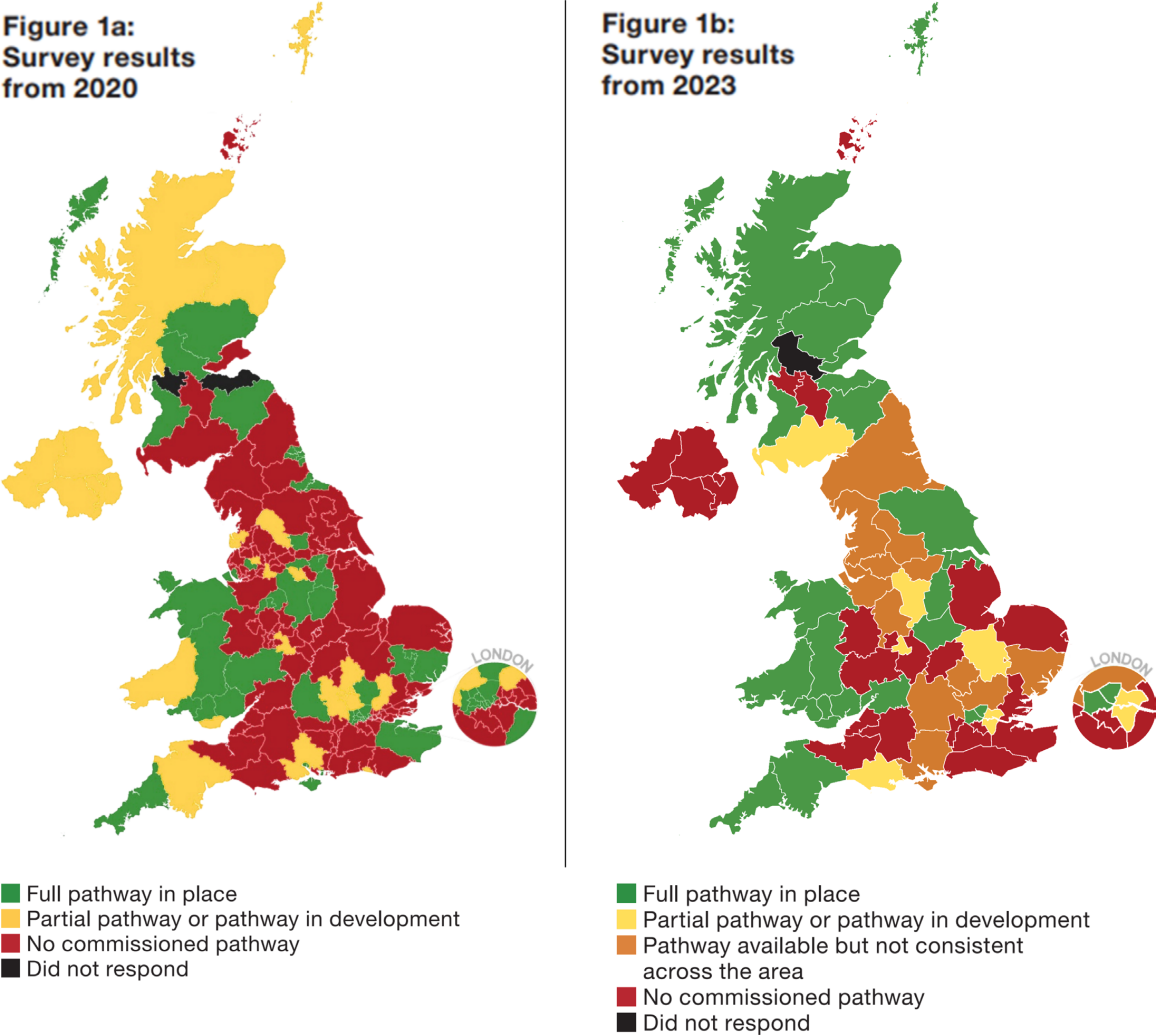
The availability of integated care systems and health board-endorsed community liver pathways in the UK. Figure 1a shows 2020 survery results; Figure 1b shows 2023 survery results.

Figure 1 provides a geographical overview of the provision of community liver pathways in the UK now compared with provision in 2020.^5^

### The use of recommended guidelines and diagnostic tools to detect liver disease

This survey also highlights increased use of some recommended liver fibrosis assessment methods, including nearly double the number of areas making use of transient elastography within community pathways of care, up from 25% to 46%. The increased use of transient elastography, as well as use of indirect serum liver fibrosis markers (up from 44% to 67%), is in contrast to the use of direct serum markers of fibrosis, that is, the ELF test, which have remained almost static. The variable and increasing use of these diagnostic methods is detailed in Table 1.

**Table 1.** The recommended management of liver disease in UK integrated care systems and health authorities in relation to national standards and guidelines

Area of UK	Using liver fibrosis assessment in a pathway, *n* (%)	Using indirect serum fibrosis markers,^a^ *n* (%)	Using direct serum fibrosis markers,^b^ *n* (%)	Using transient elastography,^c^ *n* (%)
England *n* = 42	22 (52%) **(** ***39%*** **)**	27 (64%) ***(41%*** **)**	7 (17%) **(** ***13%*** **)**	17 (40%) **(** ***19%*** **)**
Northern Ireland *n* = 5	0 (0%) ***(0%)***	1 (20%) **(** ***0%*** **)**	1 (20%) **(** ***0%*** **)**	1 (20%) **(** ***0%*** **)**
Scotland *n* = 13	9 (69%) **(** ***67%*** **)**	10 (77%) **(** ***75%*** **)**	4 (31%) **(50%)**	8 (62%) **(** ***58%*** **)**
Wales *n* = 7	7 (100%) **(** ***100%*** **)**	7 (100%) **(** ***71%*** **)**	0 (0%) **(** ***29%*** **)**	5 (71%) **(** ***100%*** **)**
UK total *n* = 67	38 (57%) **(** ***42%*** **)**	45 (67%) **(** ***44%*** **)**	12 (18%) **(** ***16%*** **)**	31 (46%) **(** ***25%*** **)**

The percentages highlighted in bold italics are the equivalent use of fibrosis tests reported in the 2020 survey^5^ ^a^Included use of Fibrosis-4 (FIB-4) score, Non-Alcoholic Fatty Liver Disease (NAFLD) Fibrosis Score and aspartate transaminase (AST):alanine transaminase (ALT) ratio (other options given). ^b^All but one response was for the enhanced liver fibrosis (ELF) blood test, single response hyaluronic acid. ^c^All using Fibroscan other than one response for shear wave ultrasound. *n* = number of commissioning bodies and health boards responding to survey

### Engagement with more proactive risk factor-based detection of liver disease

**Figure 2.**
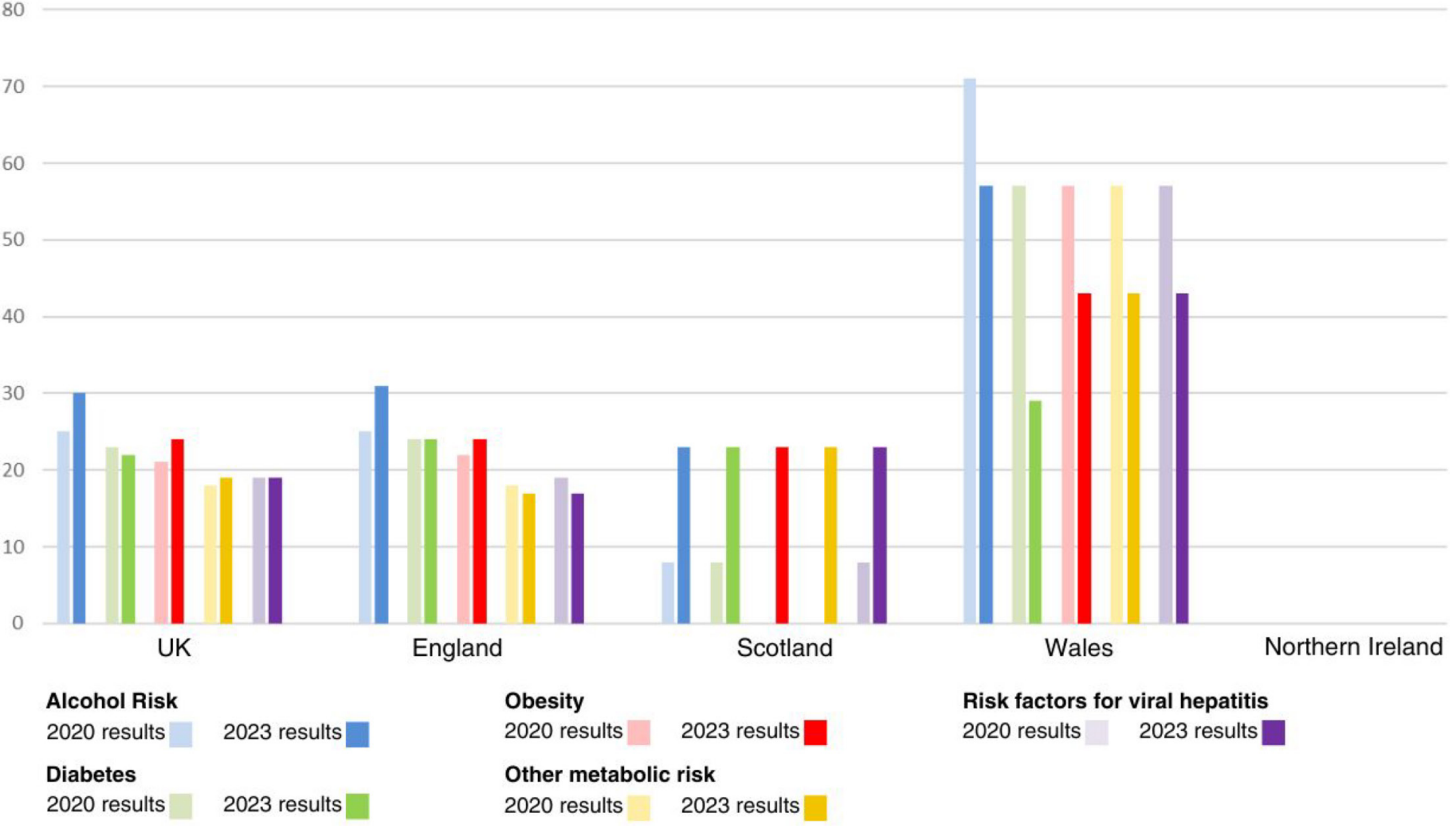
Percentage of integrated care systems and health authorities using proactive methods to identify liver disease by risk factor

**Figure 3.**
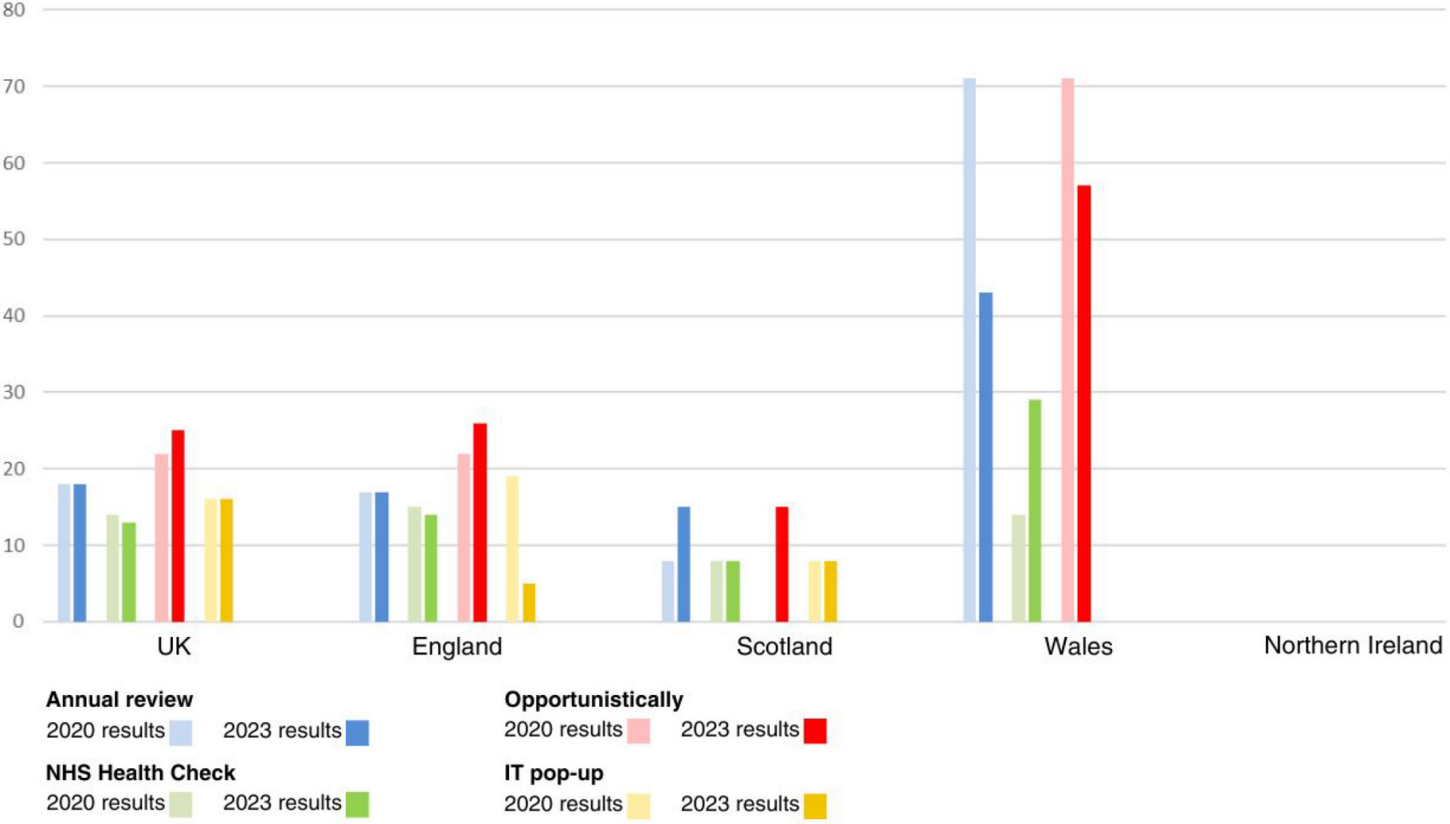
Percentage of integrated care systems and health authorities using proactive methods to identify liver disease by assessment method

There remains a minority of health boards adopting a more proactive risk factor-based approach to detect liver disease, with 21% of regions adopting such an approach. As in the previous survey, the most common risk factors taken forward for liver disease assessment were alcohol risk and diabetes. As previously, there was marked national variation in utilising this approach with it being established practice in much of Wales, with Scotland showing a marked move towards adopting this way of working also. Figures 2 and 3 depict at a national level the percentage of areas taking a risk factor-based approach (Figure 2) and how these individuals are being identified in routine primary care practice (Figure 3). The figures compare the data from the 2020 survey with the updated data.

## Discussion

### Summary

This survey provides an up-to-date national overview of commissioner and health board engagement in management of liver disease in the community.

More than one-third (36%) of areas now have comprehensive pathways of care in place, up from approximately one-quarter 3 years previously. The percentage of areas with no pathway at all in place has fallen from 58% in 2020 to 36%. In line with this increase in developing pathways of care was an increased number of areas using fibrosis testing, mainly the indirect serum markers such as FIB4 and transient elastography.

Comparing the results from the 2020 survey to responses in 2023, regional variations persist. Wales has built on outstanding results in the initial survey, with all health boards now having comprehensive pathways for care. Scotland has also seen marked improvement in its care pathways. Northern Ireland however has not managed to implement the pathways reported previously as in development,^5^ and remains without primary care pathways for liver disease.

Despite the restructuring of commissioning bodies in England from CCGs to ICS, several ICS areas were still operating at locality (old CCG) level in the commissioning of pathways. To reflect this, an additional category of provision was included when mapping the responses from England, reflecting pathways running within parts of an ICS, but not across the whole area (see Figure 1b). This makes results comparison in England more challenging.

Only 18% of areas utilised the ELF test for assessment of fibrosis, despite this being a key recommendation in the National Institute for Health and Care Excellence (NICE) guidelines for NAFLD.^11^

### Strengths and limitations

There are limitations to a cross-sectional survey approach that can only provide a snapshot view of engagement in this area. By replicating and updating a previous survey in this area, a strength of this re-survey is that progress in practice can be studied.

It is acknowledged that the survey needed to reach a responder able to answer the survey questions accurately and this limitation may have been amplified by the relatively recent formation of ICSs and boards in England. It is also acknowledged that independently of any health board or more regional recommendation, individual GPs may use their own templates based on national guidelines to manage liver disease. This variation in individual practice would be extremely difficult to capture. As clinicians work in regional environments where access to fibrosis testing is standardised, and pathways to referral involve expected algorithms to have been followed by secondary care clinicians, this is likely to limit this individual practice variation.

The study only indicates the provision of a pathway for the early detection of liver disease; it does not measure in detail how well that pathway is implemented, monitored, and evaluated.

### Comparison with existing literature

The increasing use of pathways of care to manage liver disease in the UK, indicated by this repeat commissioning engagement survey, is mirrored by findings from the secondary care provider perspective. An online survey sent initially in 2014–2015 to gastroenterology and hepatology specialists was repeated in late 2021 with a noted increase in use of fibrosis testing and reported pathways of care in place between surveys.^10^ Forty per cent of hospital trusts (represented by the response of liver specialists) had reported pathways in place in this comparator survey in 2021, which was carried out around the same time as our initial commissioners survey where we reported 26% comprehensive pathway coverage, which has now increased to 36%. The authors of the secondary care survey postulated that the difference in reported activity may partly have been owing to lack of comprehensive dissemination of available pathways of care, which is something that may have been improved between our original and current surveys.

We believe surveying health boards and commissioning bodies on engagement with liver disease management provides a unique perspective, with no directly comparable literature. There is however literature on primary care perspectives on the management of liver disease, which is mainly focused on confidence and competence in the management of MASLD (formerly known as NAFLD). Several surveys and qualitative studies from Europe and the US consistently report liver disease as an area where primary care clinicians feel they lack confidence and further guidance and education are needed.^12–15^ This evidence, suggesting widespread lack of clinical confidence, may be partly explained by the variability in system leadership highlighted as an improving but still ongoing issue in our study.

### Implications for research and practice

The change in health system structure in England towards larger areas incorporated within ICSs provides opportunity to utilise the results of this study to further drive more comprehensive pathway development. Several ICS areas have pathways in place that are still aligned to old CCG or hospital trust catchment areas, but have yet to be taken on and rolled out across the ICS area (areas highlighted as dark amber in Figure 1b). The British Liver Trust, in collaboration with academic co-authors, will be using this work to highlight this alignment and lack of rollout to integrated care board colleagues, advising on utilising and expanding on existing good practice within their regions. Published good practice examples are available on the British Liver Trust website,^16^ which can be adapted to local infrastructure and local population need.

The ongoing inequalities and inconsistencies in the way care for liver disease is commissioned in the UK highlight the need for a national health policy directive in this area. This has been achieved in Wales with a national liver disease delivery plan^17^ and we would recommend the other nations take a similar approach. This study has highlighted that even among areas with pathways for liver disease management in place, there are a variety of methods being used, both to identify patients for further testing, and in choice of non-invasive liver fibrosis test (see Table 1 and Figures 2 and 3). This reflects a still-developing evidence base in recommended liver fibrosis testing order in low prevalence community settings^18,19^ with differences within national guidelines^11,20,21^ along with more proactive approaches being advocated internationally.^22^

Ongoing research in this area is needed but should not detract from the urgent need for a central directive to mandate ICS areas and health boards to develop a locally agreed pathway of care for early detection and management of their populations at risk of liver disease, with strategies including liver fibrosis risk assessment as a minimum requirement. This is well supported by evidence that implementation of community pathways increase the detection of liver cirrhosis^23–25^ and are cost-effective.^26,27^

If the progress observed between surveys in commissioning pathways of care for liver disease is to continue, with more people being given the opportunity to be detected with disease at an earlier stage, this progress must be accompanied by increased funding to implement effective weight loss and alcohol reduction interventions in community settings as the mainstay of treatment. There is already good evidence around the short-term efficacy of such intervention strategies in reducing weight^28^ and alcohol consumption,^29^ but the availability of these interventions needs to be consistent across the country,^30^ with a need to research the longer-term health outcomes associated with good access to these interventions.

This survey provides a UK-wide overview of provision of community liver disease pathways and progress in this area since 2020. Increasing levels of engagement but ongoing regional variation should be of interest to primary care practitioners, liver specialists, and policymakers. The results of this survey should be used to further drive change and continue to reduce healthcare variation in the primary care management of chronic liver disease in the UK.

## Data

The dataset relied on in this article is available from the corresponding author on reasonable request
